# Genome-wide association study combined with multi-assay phenotyping identifies a novel anthracnose resistance locus in apple

**DOI:** 10.1186/s12870-026-09198-y

**Published:** 2026-06-24

**Authors:** Dagyeong Kwon, Baek Gyun Choi, Jong Taek Park, Seunghyun Ban, Cheol Choi

**Affiliations:** 1 Apple Research Center, National Institute of Horticultural & Herbal Science, Gunwi, 43100 Republic of Korea; 2 Department of Horticulture, College of Agriculture and Life Science, Kyungpook National University, Daegu, 41566 Republic of Korea

**Keywords:** *Malus × domestica*, Anthracnose, *Colletotrichum*, Disease resistance, Genome-wide association study, Field phenotyping, Artificial inoculation, Genotype-by-environment interaction

## Abstract

**Background:**

Apple anthracnose, a disease complex that includes *Glomerella* leaf spot (GLS) and bitter rot caused by *Colletotrichum* species, is a major disease affecting apple production worldwide. In this study, we combined multi-year field evaluations with controlled inoculation assays to identify genomic regions associated with anthracnose resistance in apple.

**Results:**

A total of 440 apple genotypes, including 411 F₁ progenies derived from six parental crosses and 29 cultivars, were evaluated under natural orchard conditions and through artificial fruit and leaf inoculation assays using wound and non-wound methods. Disease severity varied substantially between years, particularly under contrasting environmental conditions, indicating strong genotype-by-environment interactions. Genome-wide association analysis (GWAS) using field-derived disease severity scores from 2019 identified a significant quantitative trait locus (QTL) on chromosome 15 (~ 31.8 Mb) associated with reduced anthracnose severity. This locus was distinct from the previously reported *Rgls/MdTNL1* region on chromosome 15 (~ 2–5 Mb), suggesting the presence of a novel resistance-associated locus. In contrast, no genome-wide significant associations were detected from artificial inoculation datasets.

**Conclusions:**

These findings demonstrate the importance of field-based, multi-environment phenotyping for detecting field-relevant resistance loci and improving understanding of the genetic architecture underlying anthracnose resistance in apple.

**Supplementary Information:**

The online version contains supplementary material available at https://doi.org/10.1186/s12870-026-09198-y.

## Introduction

Anthracnose, caused by fungi of the *Colletotrichum* genus, is a destructive disease affecting many fruit crops worldwide [1–3]. In apple (*Malus × domestica*), *Colletotrichum* species are responsible for two major disease manifestations: bitter rot, which affects the fruit, and *Glomerella* leaf spot (GLS), which impacts the foliage. Bitter rot is caused by multiple species within the *C. acutatum* and *C. gloeosporioides* species complexes, and species within the *C. gloeosporioides* complex, including *C. fructicola*, have also been associated with *Glomerella* leaf spot on apple leaves [4–6]. Together, these anthracnose diseases have become increasingly important constraints on apple production, particularly under warm and humid environmental conditions. Bitter rot is now considered one of the most damaging fungal diseases in apple orchards, with reported fruit yield losses ranging from 14 to 25% in New York to near-total crop failures in climates highly favorable to infection [7, 8]. Similarly, GLS has emerged as a major threat in regions like Brazil, where cultivars such as ‘Gala’ are especially vulnerable [9]. The widespread distribution and severity of these pathogens underscore the urgent need for durable and sustainable control strategies.

Developing anthracnose-resistant apple cultivars is a key goal for sustainable disease management. However, most commercial apple varieties lack strong resistance to bitter rot or GLS, making breeding for resistance challenging. Notably, some apple genotypes do exhibit partial resistance; for example, ‘Fuji’ shows high field resistance to GLS, whereas ‘Gala’ and its sports are generally very susceptible [9]. Genetic studies have demonstrated that, in certain germplasm, GLS resistance can be governed by a single recessive locus, termed *Rgls* [10, 11]. The *Rgls* locus was initially mapped to apple chromosome 15 in a segregating population, and subsequent fine-mapping led to the identification of a candidate gene *MdTNL1* at this locus [12]. *Rgls*-mediated resistance has been introgressed into some new apple cultivars in Brazil, which carry *MdTNL1*-linked markers [9]. Nevertheless, some genotypes with low disease severity lack the *Rgls*-associated allele, suggesting that additional loci also contribute to anthracnose disease response.

A critical consideration in genetic studies of disease resistance is the influence of environmental variation on trait expression. Disease severity in the field is strongly affected by factors such as temperature, rainfall, and inoculum pressure, which can either obscure or amplify the resistance phenotype of a given genotype. Genotype-by-environment (G × E) interactions are often substantial for quantitative resistance traits, reducing trait heritability and altering the detectability of resistance loci across seasons or locations [13–15]. In apple anthracnose, for instance, warm and humid conditions are known to promote severe outbreaks, whereas in cooler or drier environments, even susceptible cultivars may show minimal symptom development [16]. Such environmentally driven variability complicates the accurate identification and interpretation of genetic resistance. To address these challenges, multi-environment evaluations are essential. Testing germplasm across years, locations, or under controlled inoculation conditions can help capture a more representative range of phenotypic responses [17]. In apple, disease screening has employed both natural orchard infections and artificial inoculations under controlled conditions [18]. Each approach offers distinct advantages: field assessments reflect real-world pathogen pressure and management conditions, while controlled inoculation methods, such as fruit wound inoculations or detached leaf assays, allow for standardized and replicable challenges. However, resistance rankings derived from these approaches do not always correspond. For example, studies in peach have shown only moderate correlation (*r* ≈ 0.55) between disease severity under laboratory inoculation and actual field incidence [19], illustrating the risk of misestimating resistance when phenotyping is confined to a single environment or method [17].

Building on these considerations, the present study aimed to identify genome-wide loci associated with anthracnose resistance using a multi-family F₁ population derived from controlled crosses among selected commercial and breeding apple cultivars. This experimental design enabled the detection of resistance loci segregating both within and across families, while simultaneously allowing assessment of how environmental variation influences resistance expression. By integrating multi-year field phenotyping with genome-wide association analysis, this work seeks to clarify the broader genetic architecture of anthracnose resistance beyond known major genes such as *Rgls*, and to provide field-relevant genomic insights that can inform resistance breeding strategies in apple.

## Materials and methods

### Plant materials

To establish parental disease-severity profiles for anthracnose, seven apple cultivars (‘Fuji’, ‘Tsugaru’, ‘Aikanokaori’, ‘Gala’, ‘Jonathan’, ‘Hongro’, and ‘Cripps Pink’) were evaluated under natural field infection conditions from 2009 to 2011 at the Apple Research Institute (ARI), National Institute of Horticultural & Herbal Science (NIHHS), Rural Development Administration (RDA), Gunwi, Republic of Korea. All plant materials used in this study were cultivated apple (*Malus × domestica*) accessions maintained at NIHHS, and no wild plant materials were collected. Therefore, no specific permits were required for plant material collection. The identity of all cultivars was confirmed based on institutional records and germplasm management information at NIHHS. All experimental procedures complied with relevant institutional and national guidelines for plant research. Field incidence of anthracnose was recorded annually, and cultivars were classified according to their relative resistance or susceptibility based on multi-year observations.

Based on these disease-severity profiles and breeding considerations at ARI, six biparental F₁ populations were generated in 2012 using parental combinations representing diverse anthracnose responses. The crosses included ‘Gala’ × ‘Jonathan’, ‘Gala’ × ‘Aikanokaori’, ‘Cripps Pink’ × ‘Tsugaru’, ‘Jonathan’ × ‘Gala’, ‘Hongro’ × ‘Jonathan’, and ‘Fuji’ × ‘Tsugaru’. A total of 411 F₁ progenies were produced and grafted onto M.9 rootstocks. The F₁ populations were established under standard orchard management practices recommended by the Rural Development Administration [20] and maintained without artificial inoculation during early growth stages. Anthracnose resistance phenotyping was conducted once trees reached 4–5 years of age, during the 2019 and 2020 growing seasons, when stable fruit production allowed reliable evaluation of disease incidence under natural orchard infection conditions. In total, 440 apple genotypes (411 F₁ progenies and 29 cultivars) were included in this study.

### Field incidence evaluations

Field incidence of apple anthracnose was evaluated according to the standard disease assessment protocol of the Rural Development Administration [21] (Table 1). Disease incidence was calculated as the proportion of infected fruits per tree and subsequently converted into a six-class ordinal scale, ranging from 1 (highly resistant, HR) to 6 (highly susceptible, HS). For the parental cultivars, field incidence was monitored repeatedly during the growing season, with assessments conducted weekly from July to September and biweekly from October until harvest during the 2009–2011 seasons. For the F₁ populations, field disease severity was evaluated during the 2019 and 2020 growing seasons under natural orchard infection conditions, when trees had reached stable fruit-bearing stages. The number of fruits examined per progeny ranged from 20 to 91, with an average of 31 fruits per progeny across the six populations. Longitudinal disease observations were available for parental cultivar evaluations but were not uniformly available for all F₁ progenies used in GWAS. Therefore, AUDPC was not calculated for the association panel, and final RDA degree-of-infection scores were used as GWAS phenotypes.

**Table 1:** Disease severity classification for apple anthracnose based on field incidence and artificial inoculation criteria [21]

Degree of infection	Evaluation^z^	Field incidence (%)^y^	Lesion dimension by inoculation (㎜)^x^
1	HR	0.0	0.0
2	R	< 1.0	< 1.0
3	MR	≥ 1.0 to < 5.0	≥ 1.1 to < 2.5
4	MS	≥ 5.0 to < 10.0	≥ 2.5 to < 5.0
5	S	≥ 10.0 to < 20.0	≥ 5.0 to < 10.0
6	HS	≥ 20.0	≥ 10.0

^z^ HR, Highly resistant; R, Resistant; MR, Moderately resistant; MS, Moderately susceptible; S, Susceptible; HS, Highly susceptible

^y^ Percentage of fruits showing anthracnose symptoms relative to the total number of fruits per tree

^x^ Mean lesion length calculated as the average of the longest and shortest lesion diameters following artificial inoculation

### Pathogen and inoculum preparation

A virulent isolate of *Colletotrichum gloeosporioides* (KACC 40848) was obtained from the National Agrobiodiversity Center, Rural Development Administration (RDA), Jeonju, Republic of Korea. The fungus was cultured on potato dextrose agar (PDA) plates at 25 °C for 7 days. Conidia were harvested by flooding the culture plates with sterile distilled water and gently scraping the colony surface. The resulting suspension was filtered through sterile gauze to remove mycelial debris, and the conidial concentration was adjusted to 1 × 10⁵ conidia mL⁻¹ using a hemacytometer.

### Artificial inoculation assays

Artificial inoculation assays were conducted during the 2019 and 2020 growing seasons on fruits and leaves of parental cultivars and F₁ progenies (Table 2). Fruits were collected at physiological maturity, and fully expanded leaves were sampled in mid-August. Prior to inoculation, plant materials were washed with tap water, surface-dried at room temperature, and handled under aseptic conditions. For wound inoculation on fruits, three wounds per fruit were made using a sterile pin, after which a 6-mm diameter paper disc soaked in the conidial suspension was placed onto each wound site. Paper discs were used to maintain consistent inoculum contact and localized moisture at the inoculation site during incubation. Non-wound inoculations were performed by placing inoculum discs directly onto intact fruit surfaces. For fruit inoculation assays, three to nine fruits per progeny were evaluated, with three inoculation sites applied per fruit. For leaf assays, both wound and non-wound inoculations were applied, with 30 leaves per genotype evaluated (one inoculation site per method per leaf). Inoculated fruits and leaves were incubated in sealed plastic containers at 28 °C under near-saturated relative humidity (~ 100%) with a 12-h photoperiod. Inoculated materials from different genotypes were randomly positioned within incubation containers during disease development to minimize positional effects. Lesion development was assessed 10 days after inoculation, and lesion size was measured as the mean of the longest and shortest diameters. Lesion measurements were converted to the six-class RDA disease severity scale.

**Table 2:** Distribution of apple genotypes evaluated for anthracnose resistance under field and controlled inoculation conditions (2019–2020)

Population/Cultivars (No.)	Field incidence		Inoculation in vitro			
	Fruit		Fruit (wound)		Leaf (wound and non-wound)	
	2019	2020	2019	2020	2019	2020
Gala × Jonathan (180)	162	175	64	52	172	172
Gala × Aikanokaori (71)	45	45	7	33	0	0
Cripps pink × Tsugaru (60)	60	60	14	0	0	0
Jonathan × Gala (35)	34	34	6	16	0	0
Hongro × Jonathan (33)	3	3	0	32	0	0
Fuji × Tsugaru (32)	32	32	5	0	0	0
Cultivars^z^ (29)	4	4	4	4	29	29
Total (440)	340	353	100	137	201	201

^Z^Cultivars included ‘Fuji’, ‘Gala’, ‘Hongro’, ‘Jonathan’, ‘Alpsottome’, ‘Arione’, ‘Arisoo’, ‘Buddybell’, ‘Charmingbell’, ‘Colorpple’, ‘Easypple’, ‘Gamhong’, ‘Goldenball’, ‘Grannysmith’, ‘Greenball’, ‘Hanabell’, ‘Honeycrisp’, ‘Hongguem’, ‘Hwangok’, ‘Orin’, ‘Picnic’, ‘Ruby-S’, ‘Shinanogold’, ‘Summerdream’, ‘Summerking’, ‘Summerprince’, ‘Yoko’, ‘Cripps Pink’, and ‘Tsugaru’

### Statistical analysis

Phenotypic data were compiled and summarized in spreadsheet format, and statistical analyses were performed with SPSS version 21.0 (IBM Corp., Chicago, IL, USA). Pearson’s correlation coefficients were calculated to assess relationships between field incidence and artificial inoculation responses. Differences among cultivar means were evaluated using Duncan’s multiple range test, with statistical significance declared at *p* < 0.05.

### Heritability estimation

Broad-sense heritability ($\:{H}^{2}$) was estimated separately for field disease severity and controlled fruit inoculation traits using combined disease severity scores obtained in 2019 and 2020. A linear mixed model was fitted using the lme4 [22] package in R, with year treated as a fixed effect and genotype accession treated as a random effect:

$$\:{y}_{ij}=\:\mu\:+\:{Y}_{i}+\:{G}_{j}+\:{e}_{ij}$$

Where $\:{y}_{ij}$ is the phenotypic value, $\:\mu\:$ is the overall mean, $\:{Y}_{i}$ is the fixed effect of year, $\:{G}_{j}$ is the random effect of genotype accession, and $\:{e}_{ij}$ is the residual error.

Broad-sense heritability was calculated as:

$$\:{H}^{2}=\frac{{\sigma\:}_{g}^{2}}{{\sigma\:}_{g}^{2}+\frac{{\sigma\:}_{e}^{2}}{r}}$$

Where $\:{\sigma\:}_{g}^{2}$ is the genotypic variance, $\:{\sigma\:}_{e}^{2}$ is the residual variance, and * r* is the average number of observations per genotype.

### DNA extraction and GBS library preparation

Genomic DNA was extracted from young leaves of 440 apple samples (411 F₁ progenies and 29 cultivars) using the DNeasy Plant Mini Kit (Qiagen, Germany) according to the manufacturer’s instructions. DNA quality and quantity were assessed using spectrophotometric measurements (A260/280 and A260/230 ratios), agarose gel electrophoresis, and Quant-iT™ PicoGreen^®^ dsDNA assays.

Genotyping-by-sequencing (GBS) libraries were prepared following a modified ApeKI-based protocol. Briefly, 250 ng of genomic DNA per sample was digested with ApeKI (New England BioLabs, USA) at 75 °C for 3 h, followed by ligation of barcoded adapters (4–9 bp unique barcodes per sample) using T4 DNA ligase. Up to 96 barcoded samples were pooled per library, purified using the NucleoSpin^®^ Gel and PCR Clean-up kit (Macherey-Nagel), and amplified by multiplex PCR with Illumina index primers. PCR products were size-selected (400–700 bp) using SPRIselect beads, and library quality was assessed with an Agilent 4200 TapeStation. Sequencing was performed on an Illumina NovaSeq 6000 platform to generate 151 bp paired-end reads.

### Read processing and SNP discovery

Raw sequencing reads were demultiplexed using STACKS v2.60 [23] (process_radtags) and trimmed with Trimmomatic v0.39 [24] to remove adapter sequences and low-quality bases. High-quality reads were aligned to the *Malus × domestica* HFTH1 reference genome using BWA-MEM v0.7.17 [25]. Resulting BAM files were processed with Picard [26] tools to assign read group information and remove technical artifacts. Variant calling was conducted using GATK [27] HaplotypeCaller in GVCF mode, followed by joint genotyping with GenotypeGVCFs. Raw variants were filtered to retain bi-allelic SNPs only and further screened using the following criteria: QUAL ≥ 30, QD ≥ 5, FS ≤ 200, mean read depth ≥ 5, minor allele frequency (MAF) ≥ 0.05, and missing data ≤ 30%. Filtering was performed using GATK VariantFiltration and VCFtools v0.1.16 [28]. After quality control, a total of 76,080 high-quality SNPs were retained for downstream association analysis.

### Population structure and kinship analysis

Principal component analysis (PCA) and phylogenetic analysis were conducted to evaluate population structure among the F₁ progenies and parental cultivars using the final filtered SNP dataset. PCA was performed using genome-wide SNP data implemented in GAPIT [29] through the ‘prcomp()’ function in R. Different numbers of principal components were evaluated during model optimization, and the first five principal components were ultimately selected for GWAS analyses.

A neighbor-joining tree was constructed using Tassel 5.0 [30] software based on SNP-derived genetic distances to visualize genetic relationships among F₁ progenies and parental cultivars. The phylogenetic tree was constructed using Tassel 5.0 software and exported in Newick format. Genomic relatedness among individuals was further estimated using a kinship matrix (K) calculated with the VanRaden method implemented in the rrBLUP [31] R package.

### Genome-wide association analysis

GWAS was conducted using the GAPIT R package, employing a combination of single-locus (MLM) and multi-locus mixed models (MLMM, FarmCPU, BLINK, and SUPER). Population structure and genetic relatedness were controlled using principal component covariates (Q) and a kinship matrix derived from the analyses described above. The first five principal components were included as covariates in GWAS models to account for population structure. Statistical significance was determined using a Bonferroni-adjusted threshold (*p* < 0.05 / number of SNPs). For GWAS, phenotypic data derived from both field evaluations and artificial inoculation assays were analyzed. Field-derived phenotypes were analyzed as ordinal degree-of-infection scores on a 1–6 scale, converted from field incidence according to RDA criteria. Inoculation-based traits were analyzed using the corresponding disease severity scores derived from lesion measurements. Phenotypic datasets used for GWAS are provided as Supplementary Table S1 and phenotypic distributions of the disease severity scores used for GWAS are shown in Supplementary Figure S1. GWAS results were visualized using Manhattan plot. To further assess the robustness of the GAPIT-based results, supplementary GWAS analyses were performed using GEMMA [32], implementing a linear mixed model (LMM) with the same SNP dataset, phenotype data, principal component covariates, and kinship matrix used in the GAPIT analyses.

## Results

### Parental cultivars show contrasting field disease severity

Anthracnose incidence was assessed in seven apple cultivars over three consecutive growing seasons under natural field conditions. Disease incidence varied among years, but clear cultivar-level differences were observed (Table 3). ‘Fuji’, ‘Aikanokaori’, ‘Tsugaru’, and ‘Gala’ consistently showed low mean disease incidence at or below 1.0%, whereas ‘Jonathan’, ‘Hongro’, and ‘Cripps Pink’ showed higher mean incidence values of 15.1%, 22.7%, and 27.6%, respectively. These multi-year field evaluations established contrasting parental disease-severity profiles for subsequent population development.

**Table 3:** Field disease incidence and RDA degree-of-infection class of parental cultivars from 2009 to 2011

Cultivar	Incidence by year (%)				Degree-of-infection class
	2009	2010	2011	Average	
Fuji	0	0.6	0	0.2 a^z^	Resistant
Aikanokaori	1.7	0	0	0.6 a	Resistant
Tsugaru	0	2.1	0	0.7 a	Resistant
Gala	0.7	0	2.3	1.0 a	Moderately resistant
Jonathan	25.1	15.0	5.1	15.1 b	Susceptible
Hongro	35.2	28.1	4.9	22.7 c	Highly susceptible
Cripps Pink	28.8	32.1	21.9	27.6 c	Highly susceptible

^Z^ Means followed by the same letter are not significantly different according to Duncan’s multiple range test, *p <* 0.05

### Field disease severity shifts toward higher scores under 2020 disease pressure

Field disease severity was evaluated in six biparental F₁ populations over two consecutive seasons using fruit incidence under natural orchard infection (Table 4). Disease severity shifted toward higher scores in 2020: the lowest disease-severity class decreased from 30.4% in 2019 to 19.2% in 2020, whereas the combined higher-score classes increased from 68.1% to 77.8%. This shift indicates a strong year effect on disease expression.

**Table 4:** Distribution of field-derived RDA disease severity classes in six F₁ populations from 2019 to 2020

Evaluation ^z^	No. progenies ^y^ (Ratio, %)						
	Total	G x J	C x T	G x A	J x G	H x J	F x T
				*2019*			
HR	102 (30.4)	65 (40.1)	20 (33.3)	3 (6.7)	5 (14.7)	0 (0)	9 (28.1)
R	0 (0)	0 (0)	0 (0)	0 (0)	0 (0)	0 (0)	0 (0)
MR	5 (1.5)	3 (1.9)	1 (1.7)	0 (0)	0 (0)	0 (0)	1 (3.1)
MS	26 (7.7)	8 (4.9)	11 (18.3)	1 (2.2)	1 (2.9)	0 (0)	5 (15.6)
S	48 (14.3)	18 (11.1)	8 (13.3)	7 (15.6)	13 (38.2)	1 (33.3)	1 (3.1)
HS	155 (46.1)	68 (42)	20 (33.3)	34 (75.6)	15 (44.1)	2 (66.7)	16 (50)
Total	336	162	60	45	34	3	32
				*2020*			
HR	67 (19.2)	25 (14.3)	22 (36.7)	2 (4.4)	5 (14.7)	0 (0)	13 (40.6)
R	0 (0)	0 (0)	0 (0)	0 (0)	0 (0)	0 (0)	0 (0)
MR	10 (2.9)	7 (4)	1 (1.7)	1 (2.2)	0 (0)	0 (0)	1 (3.1)
MS	34 (9.7)	19 (10.9)	7 (11.7)	2 (4.4)	2 (5.9)	0 (0)	4 (12.5)
S	56 (16)	37 (21.1)	8 (13.3)	5 (11.1)	2 (5.9)	0 (0)	4 (12.5)
HS	182 (52.1)	87 (49.7)	22 (36.7)	35 (77.8)	25 (73.5)	3 (100)	10 (31.3)
Total	349	175	60	45	34	3	32

^z^ HR, Highly resistant; R, Resistant; MR, Moderately resistant; MS, Moderately susceptible; S, Susceptible; HS, Highly susceptible

^y^ G x J, ‘Gala’ x ‘Jonathan’; C x T, ‘Cripps Pink’ x ‘Tsugaru’; G x A, ‘Gala’ x ‘Aikanokaori’; J x G, ‘Jonathan’ x ‘Gala’; H x J, ‘Hongro’ x ‘Jonathan’; F x T, ‘Fuji’ x ‘Tsugaru’

At the population level, most crosses showed a similar shift toward higher disease severity in 2020, although the magnitude of the shift differed among families (Table 4).

Weather conditions likely contributed to the observed differences in resistance class distributions between years. According to the 2020 Climate Change Monitoring Comprehensive Analysis Report issued by the Korea Meteorological Administration (2021), total summer precipitation in 2020 reached 142.7% of the 1991–2020 climatological normal, and the rainy season persisted for 28.5 days, the longest duration recorded in the past decade. In particular, June–August rainfall was substantially higher in 2020 than in 2019 at Gunwi (approximately 798 vs. 406 mm), likely increasing infection pressure and shifting field disease scores upward.

### Fruit and leaf assays reveal tissue- and method-dependent responses

Anthracnose disease responses were evaluated in 29 apple cultivars using field observations and controlled inoculation assays (Table 5). Four cultivars were assessed under both orchard and laboratory conditions, whereas the remaining 25 cultivars were evaluated using leaf inoculation assays.

**Table 5:** Field incidence and lesion-size-based disease severity of 29 apple cultivars evaluated under field and controlled inoculation conditions from 2019 to 2020

Cultivar	Fruit				Leaf			
	Field incidence		Wound inoculation		Wound inoculation		Non-wound inoculation	
	2019	2020	2019	2020	2019	2020	2019	2020
Fuji	HR(1.8)	R(0)	MR(1.90)	MR(2.39)	MR(1.16)	R(0.73)	HR(0)	HR(0)
Gala	MR(4.9)	MR(2.8)	MR(2.33)	MR(2.36)	HS(16.74)	MS(4.01)	HS(11.84)	HR(0)
Hongro	S(11.4)	S(13.1)	HS(11.2)	HS(20.5)	MR(1.89)	HR(0)	HR(0)	HR(0)
Jonathan	S(18.0)	S(15.0)	S(8.61)	S(6.48)	S(9.82)	HR(0)	S(5.50)	MR(2.02)
Alpsottome	-	-	-	-	HR(0)	HR(0)	HR(0)	HR(0)
Arione	-	-	-	-	S(8.22)	HS(11.2)	R(0.72)	MR(1.49)
Arisoo	-	-	-	-	S(7.34)	HS(14.22)	HR(0)	S(5.93)
Buddybell	-	-	-	-	MR(1.69)	S(7.64)	HR(0)	S(6.61)
Charmingbell	-	-	-	-	MR(1.99)	S(9.60)	S(5.05)	MS(4.47)
Colorpple	-	-	-	-	MS(2.85)	MR(2.47)	HR(0)	MR(1.12)
Easypple	-	-	-	-	HS(11.36)	MS(4.07)	MR(2.30)	R(0.21)
Gamhong	-	-	-	-	MR(2.45)	HS(13.46)	HR(0)	MR(2.09)
Goldenball	-	-	-	-	S(7.45)	HR(0)	HR(0)	R(0.50)
Grannysmith	-	-	-	-	R(0.38)	MR(1.10)	HR(0)	R(0.24)
Greenball	-	-	-	-	HS(11.83)	HS(10.68)	HR(0)	MS(2.60)
Hanabell	-	-	-	-	S(6.26)	HS(23.32)	S(6.05)	HS(10.72)
Honeycrisp	-	-	-	-	HS(10.79)	HS(11.63)	S(5.50)	HR(0)
Honggeum	-	-	-	-	MS(4.04)	MS(4.64)	MR(1.13)	HR(0)
Hwangok	-	-	-	-	MR(1.96)	HR(0)	HR(0)	HR(0)
Orin	-	-	-	-	MR(1.57)	HR(0)	MR(1.35)	HR(0)
Picnic	-	-	-	-	R(0.82)	MR(2.29)	HR(0)	HR(0)
Cripps Pink	-	-	-	-	HS(20.25)	HR(0)	HS(19.11)	HR(0)
Ruby-S	-	-	-	-	S(6.61)	HS(11.64)	MR(1.01)	S(7.36)
Shinanogold	-	-	-	-	HR(0)	R(0.21)	HR(0)	MR(1.92)
Summerdream	-	-	-	-	HS(11.22)	HS(16.12)	HS(11.0)	S(5.81)
Summerking	-	-	-	-	MS(4.23)	MR(1.76)	HR(0)	MR(2.34)
Summerprince	-	-	-	-	MS(4.33)	R(0.95)	MR(1.15)	HR(0)
Tsugaru	-	-	-	-	HR(0)	R(0.72)	HR(0)	HR(0)
Yoko	-	-	-	-	HS(20.27)	HS(14.98)	HS(11.0)	R(0.89)

Values in parentheses indicate field incidence (%) for field evaluations and mean lesion diameter (mm) for controlled inoculation assays. HR, R, MR, MS, S, and HS indicate RDA degree-of-infection classes defined in Table 1

Fruit-based field and wound-inoculation evaluations showed broadly similar trends among the four overlapping cultivars. In contrast, leaf responses varied substantially depending on cultivar, year, tissue type, and inoculation method. Wound inoculation generally produced higher disease severity scores than non-wound inoculation, indicating that inoculation method strongly influenced phenotypic classification.

### Wound fruit inoculation produces broader disease-severity variation

To complement the field observations, the six F₁ populations were evaluated by artificial fruit inoculation with Colletotrichum in 2019 and 2020 (Table 6). A total of 96 progeny fruits were inoculated in 2019 and 133 in 2020 across the six populations, with the ‘Gala’ × ‘Jonathan’ cross contributing the largest number of fruits in both years (2019: *n* = 64; 2020: *n* = 52), while the remaining crosses were represented by smaller sample sizes.

**Table 6:** Distribution of fruit lesion-based RDA disease severity classes in six F₁ populations after wound inoculation from 2019 to 2020

Evaluation ^z^	No. progenies (Ratio, %)						
	Total	G x J	C x T	G x A	J x G	H x J	F x T
				*2019*			
HR	22 (22.9)	15 (23.4)	5 (35.7)	0 (0)	2 (33.3)	0 (0)	0 (0)
R	1 (1.0)	1 (1.6)	0 (0)	0 (0)	0 (0)	0 (0)	0 (0)
MR	5 (5.2)	5 (7.8)	0 (0)	0 (0)	0 (0)	0 (0)	0 (0)
MS	24 (25.0)	16 (25.0)	3 (21.4)	2 (28.6)	2 (33.3)	0 (0)	1 (20.0)
S	27 (28.1)	15 (23.4)	4 (28.6)	4 (57.1)	1 (16.7)	0 (0)	3 (60.0)
HS	17 (17.7)	12 (18.8)	2 (14.3)	1 (14.3)	1 (16.7)	0 (0)	1 (20)
Total	96	64	14	7	6	0	5
				*2020*			
HR	30 (22.6)	22 (42.3)	0 (0)	2 (6.1)	5 (31.3)	1 (3.1)	0 (0)
R	10 (7.5)	5 (9.6)	0 (0)	2 (6.1)	3 (18.8)	0 (0)	0 (0)
MR	9 (6.8)	5 (9.6)	0 (0)	0 (0)	4 (25.0)	0 (0)	0 (0)
MS	22 (16.5)	7 (13.5)	0 (0)	10 (30.3)	3 (18.8)	2 (6.3)	0 (0)
S	24 (18.0)	6 (11.5)	0 (0)	12 (36.4)	1 (6.3)	5 (15.6)	0 (0)
HS	38 (28.6)	7 (13.5)	0 (0)	7 (21.2)	0 (0)	24 (75.0)	0 (0)
Total	133	52	0	33	16	32	0

^z^ HR, Highly resistant; R, Resistant; MR, Moderately resistant; MS, Moderately susceptible; S, Susceptible; HS, Highly susceptible

Artificial fruit inoculation showed clear method-dependent differences in disease expression (Table 6). Non-wound inoculation produced minimal symptom development across both years and therefore had limited ability to distinguish among genotypes. In contrast, wound inoculation produced broader variation in lesion severity, with most progenies falling into intermediate to high disease-severity classes. These results indicate that wounding improved phenotypic discrimination in fruit inoculation assays.

### Leaf inoculation responses differ between wound and non-wound assays

Leaf inoculation assays in the ‘Gala’ × ‘Jonathan’ population also showed method-dependent differences in disease severity (Table 7). Wound inoculation produced broader disease-severity variation, whereas non-wound inoculation shifted the distribution toward lower disease-severity scores in both years. These results further indicate that inoculation method strongly affects disease-score distributions in controlled assays.

**Table 7:** Distribution of leaf lesion-based RDA disease severity classes in the ‘Gala’ × ‘Jonathan’ population after wound and non-wound inoculation from 2019 to 2020

Evaluation ^z^	No. progenies (Ratio, %)			
	Wound inoculation		Non-wound inoculation	
	2019	2020	2019	2020
HR	63 (36.6)	76 (44.2)	125 (72.7)	88 (51.2)
R	37 (21.5)	22 (12.8)	17 (9.9)	19 (11.0)
MR	30 (17.4)	33 (19.2)	17 (9.9)	35 (20.3)
MS	22 (12.8)	19 (11.0)	5 (2.9)	16 (9.3)
S	15 (8.7)	13 (7.6)	6 (3.5)	8 (4.7)
HS	5 (2.9)	9 (5.2)	2 (1.2)	6 (3.5)
Total	172	172	172	172

^z^ HR, Highly resistant; R, Resistant; MR, Moderately resistant; MS, Moderately susceptible; S, Susceptible; HS, Highly susceptible

### Correlation analysis and heritability of disease severity traits

Disease severity scores varied among cultivars across field and fruit wound evaluations conducted in 2019 and 2020. Field disease severity showed a moderate positive correlation between years (*r* = 0.44, *p* < 0.05). In contrast, correlations between field and fruit wound evaluations were weak (*r* = − 0.14 to 0.06, *p* > 0.05), and fruit wound evaluations between years also showed a weak correlation (*r* = 0.19, *p* > 0.05). Broad-sense heritability (H²) for combined 2019 and 2020 field disease severity was estimated at 0.628, indicating that genetic factors substantially contributed to variation in field disease scores despite environmental influences. In contrast, the controlled fruit inoculation assay showed low heritability (H² = 0.078).

### Genotyping-by-sequencing and SNP dataset construction

Genotyping-by-sequencing (GBS) was performed on a total of 440 samples (Table 2), generating approximately 12.1 billion bases of high-quality sequencing reads that were aligned to the *Malus × domestica* HFTH1 reference genome [33]. On average, 241,205 reads per sample were successfully mapped, resulting in a mean sequencing depth of 8.38× and a median depth of 2.87×. These data corresponded to an average genome coverage of 7.33% across samples. Because GBS is a reduced-representation sequencing approach, this value reflects the proportion of the reference genome covered by *ApeKI*-associated reads rather than whole-genome sequencing coverage.

**Fig. 1:**
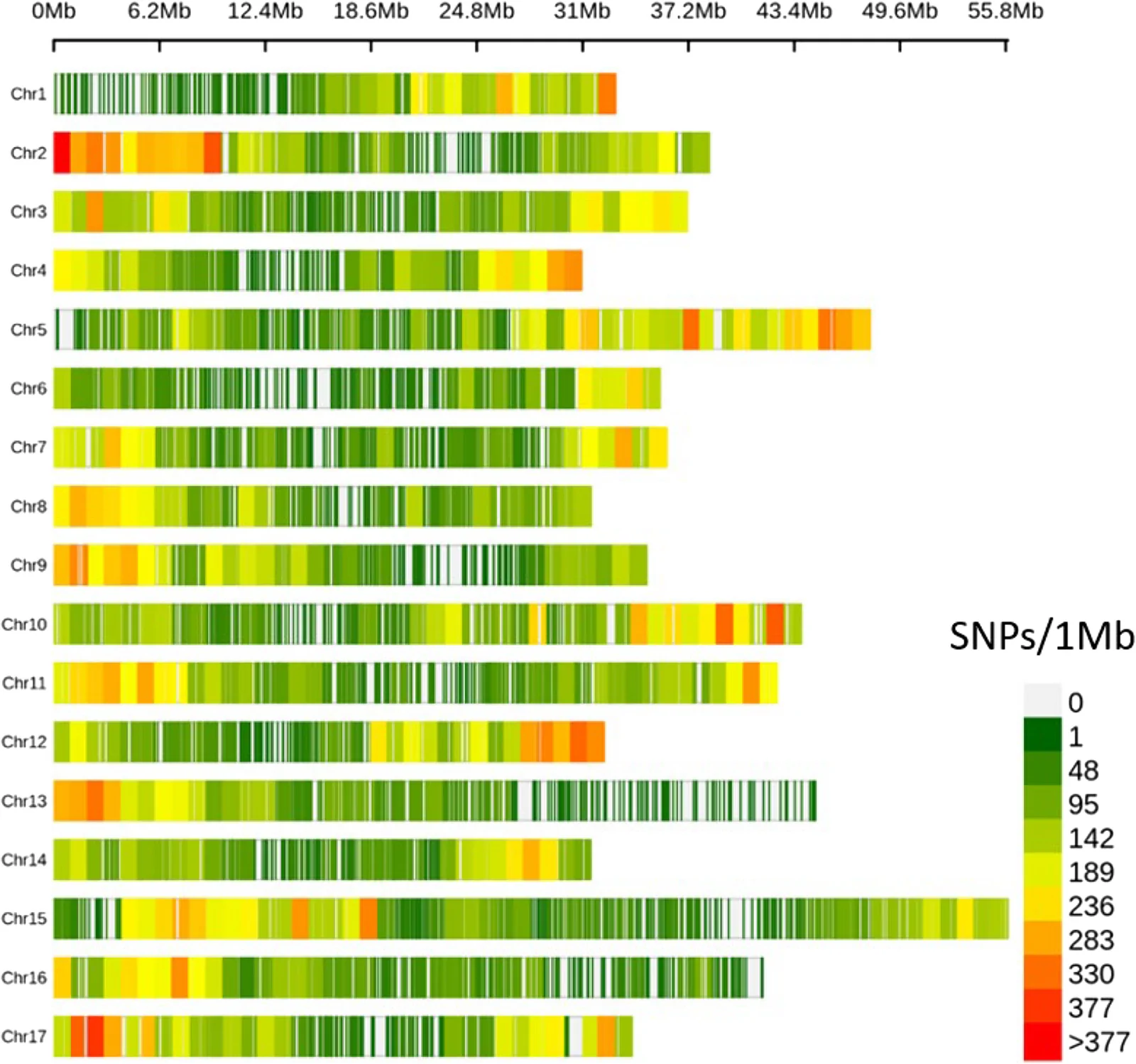
Genome-wide distribution of 76,080 filtered SNPs derived from genotyping-by-sequencing across the 17 apple chromosomes, shown as SNP density per 1 Mb window

A total of 2,154,632 raw SNPs were initially identified. After filtering for minor allele frequency (MAF > 0.05), missing data (< 30%), and bi-allelic SNPs, 76,080 high-quality SNPs were retained for GWAS analysis. These SNPs were distributed across all 17 apple chromosomes, providing broad genome-wide coverage for association mapping (Fig. 1). This filtered SNP set was subsequently used for all GWAS analyses to identify loci associated with anthracnose resistance.

### Population structure and kinship analysis for GWAS

To evaluate population structure and genetic relationships among the analyzed genotypes, phylogenetic analysis, principal component analysis (PCA), and kinship estimation were performed using the final filtered SNP dataset.

**Fig. 2:**
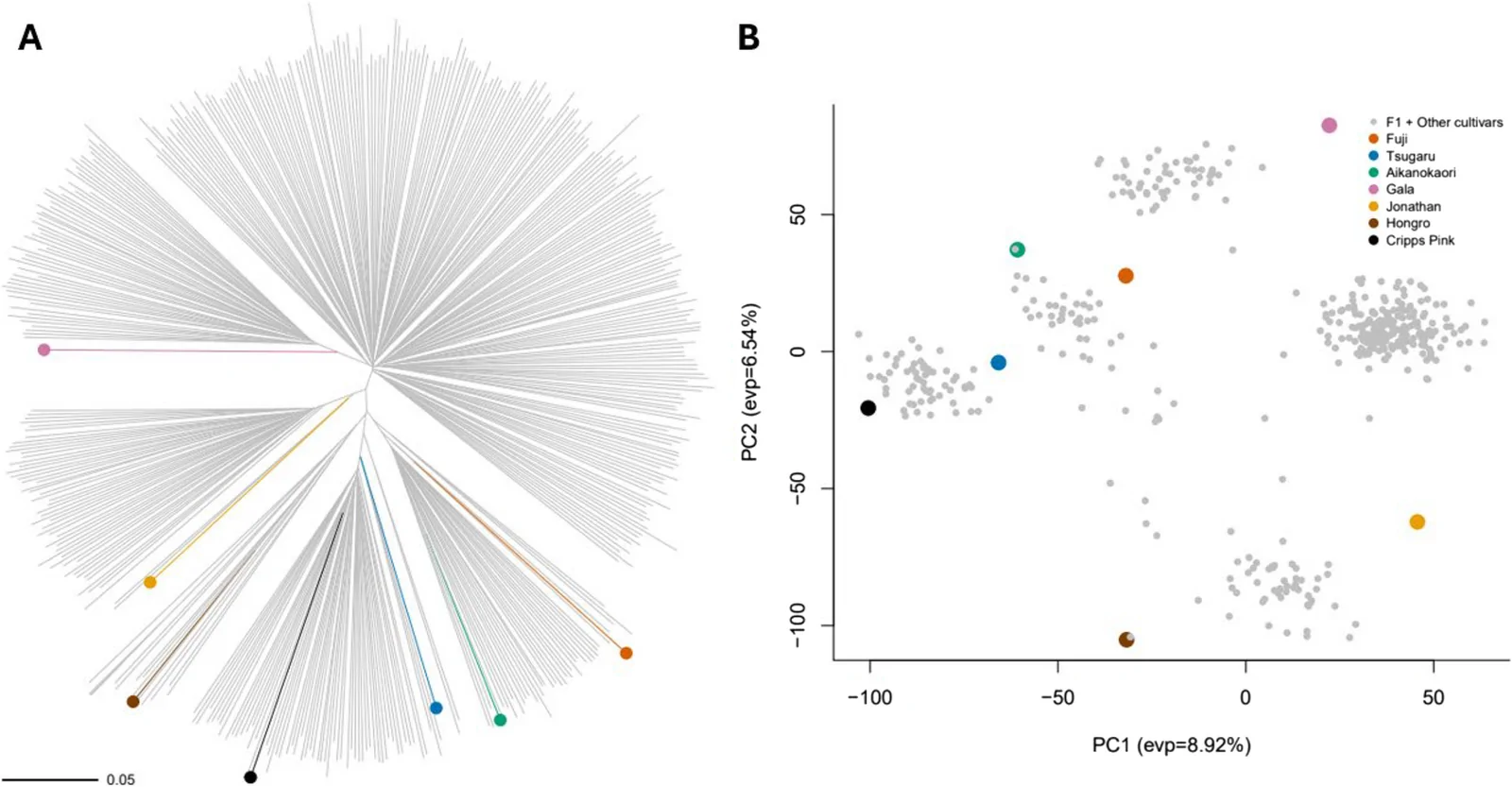
Population structure and genetic relationship analyses of the analyzed apple genotypes based on 76,080 genome-wide SNPs. **A** Neighbor-joining phylogenetic tree showing genetic relationships among parental cultivars, F₁ progenies, and additional accessions included in the analysis. Colored branches and points indicate parental cultivars, while gray branches represent the remaining genotypes. **B** Principal component analysis (PCA) plot of the analyzed genotypes. Parental cultivars are highlighted in color, whereas the remaining genotypes are shown in gray. The first two principal components explained 8.92% and 6.54% of the total genetic variation, respectively

Phylogenetic analysis based on 76,080 genome-wide SNPs showed clear genetic differentiation among the parental cultivars, while the F₁ progenies were broadly distributed throughout the tree structure with partial clustering according to pedigree background (Fig. 2A). However, substantial intermixing among progenies from different biparental populations was also observed, indicating that the analyzed populations were not completely genetically isolated.

PCA further supported the phylogenetic results, showing that parental cultivars occupied distinct positions within the genetic space, whereas the F₁ progenies were distributed across intermediate and partially overlapping regions between parental backgrounds (Fig. 2B). The first two principal components explained 8.92% and 6.54% of the total genetic variation, respectively, together accounting for 15.46% of the total genetic variation. Although partial grouping patterns reflecting pedigree structure were observed, extensive overlap among progenies suggested moderate rather than strong population stratification.

Pairwise kinship estimation revealed variable levels of relatedness among individuals, reflecting the complex pedigree structure generated from multiple biparental populations. Higher relatedness was generally observed within biparental families, whereas broader overlap among populations indicated moderate overall population structure. These results supported the inclusion of both principal component and kinship corrections in subsequent GWAS analyses.

### Genome-wide association analysis

#### Field-derived disease scores identify the most robust chromosome 15 association

**Fig. 3:**
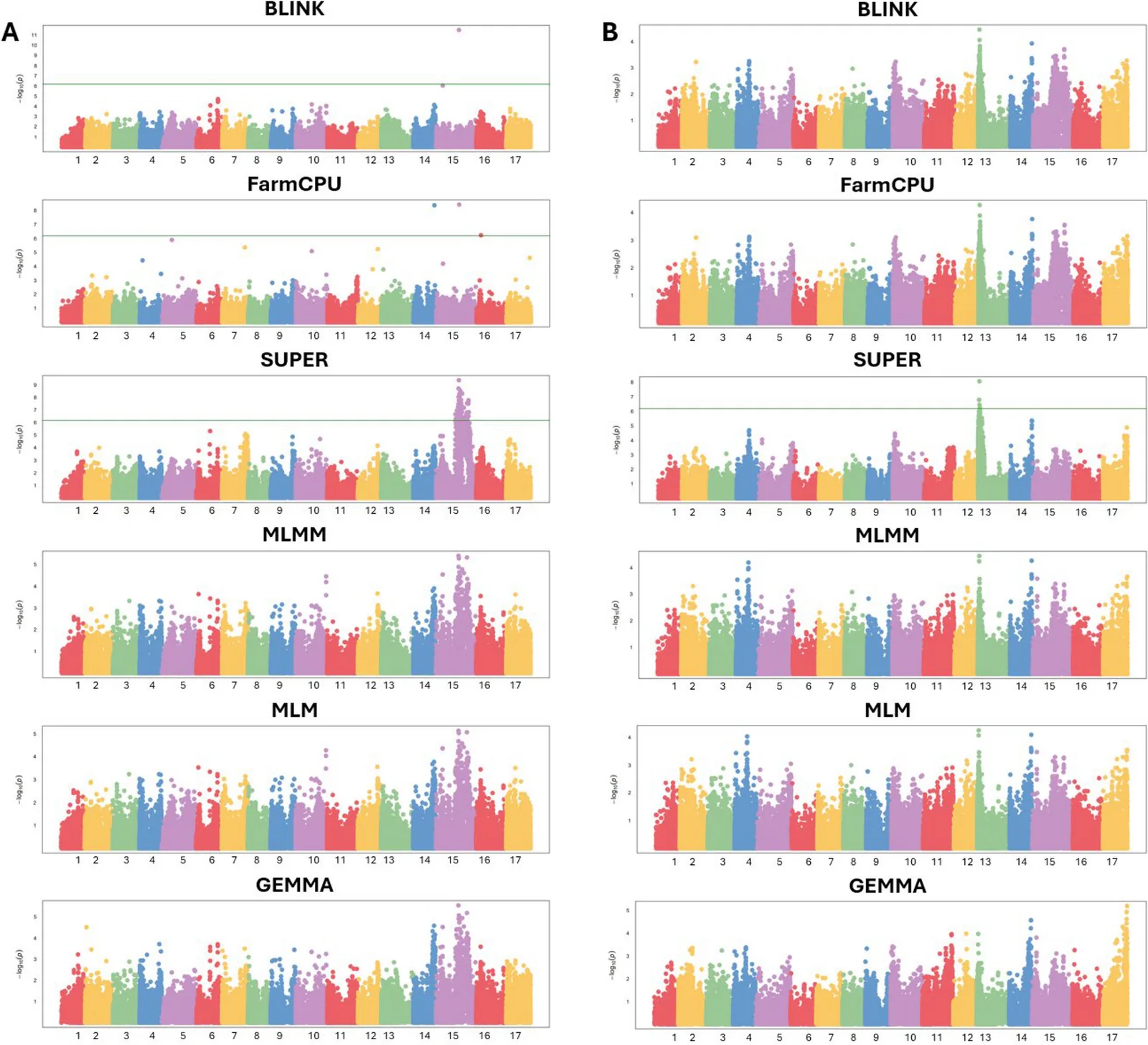
Genome-wide association study (GWAS) results for field-derived anthracnose disease severity scores in apple, expressed as degree-of-infection scores on a 1–6 scale, across six F₁ populations and four parental cultivars in 2019 (**A**) and 2020 (**B**), analyzed using five GAPIT models (BLINK, FarmCPU, SUPER, MLMM, and MLM) and one GEMMA model (LMM). The solid green line indicates the Bonferroni-corrected genome-wide significance threshold

Genome-wide association studies (GWAS) were conducted to identify loci associated with anthracnose resistance using phenotypic data obtained under naturally occurring field infection in 2019 and 2020. Genome-wide significant marker–trait associations were detected exclusively from field disease ratings (degree of infection; 1–6 scale), whereas no SNPs exceeded the significance threshold in any artificial inoculation assays (fruit or leaf). All reported associations surpassed the Bonferroni-corrected genome-wide significance threshold (*p* < 0.05 / 76,080 SNPs; –log₁₀*p* ≈ 6.2) and are summarized in Table 8. Manhattan plots for each year are shown in Fig. 3. Supplementary GEMMA analysis produced broadly similar genome-wide association patterns to the GAPIT-based models, although no additional major association peaks were detected.

**Table 8:** Summary of genome-wide significant SNPs associated with field-derived anthracnose disease severity scores in apple

Chr.	Position (bp)	Ref.	Alt.	-Log (*p*-value)	Genic/ Intergenic	Feature	Gene	Description	Model
*(Six populations and four cultivars – 2019 field degree-of-infection score)*									
14	28,904,135	C	G	8.37	Intergenic	-	DVH24_023685	Probable galacturonosyltransferase 11	FarmCPU
15	31,830,551	G	C	11.51(B) 8.42 (F)	Intergenic	-	DVH24_021233	ATP-dependent 6-phosphofructokinase 5	BLINK / FarmCPU
16	6,076,578	A	T	6.23	Genic	Exon, CDS	DVH24_007057	Uncharacterized protein	FarmCPU
*(Six populations and four cultivars – 2020 field degree-of-infection score)*									
13	1,540,751	C	G	6.95	Genic	Exon, CDS	DVH24_040330	DELLA protein GAIP-B	SUPER
13	1,541,054	A	T	6.95	Genic	Exon, CDS	DVH24_040330	DELLA protein GAIP-B	SUPER
13	2,003,851	C	G	8.06	Genic	Intron	DVH24_040384	Vacuolar-sorting protein BRO1	SUPER
13	2,173,948	A	T	6.45	Genic	Exon, CDS	DVH24_040406	Zinc finger CCCH domain-containing protein 15	SUPER
13	2,593,096	C	G	6.24	Intergenic	-	DVH24_040451	RNA-dependent RNA polymerase 1	SUPER
*(‘Gala’ x ‘Jonathan’ population – 2020 field degree-of-infection score)*									
10	3,273,660	G	C	7.54	Genic	Intron	DVH24_009327	Putative pentatricopeptide repeat-containing protein At3g01580	FarmCPU
14	29,200,202	A	T	9.41	Intergenic	-	DVH_023644	Peptidyl-prolyl cis-trans isomerase CYP26-2	FarmCPU
15	9,206,118	T	A	9.19(B) 6.25(M)	Genic	Intron	DVH24_012104	Protein WVD2-like 5	BLINK, MLMM
15	47,005,021	G	C	7.37	Genic	Intron	DVH24_016791	Phospho-2-dehydro-3-deoxyheptonate aldolase 1	BLINK
16	7,195,414	G	C	8.60	Intergenic	Exon, CDS	DVH24_033699	Uncharacterized protein	FarmCPU

### GWAS results from the 2019 field dataset

**Fig. 4:**
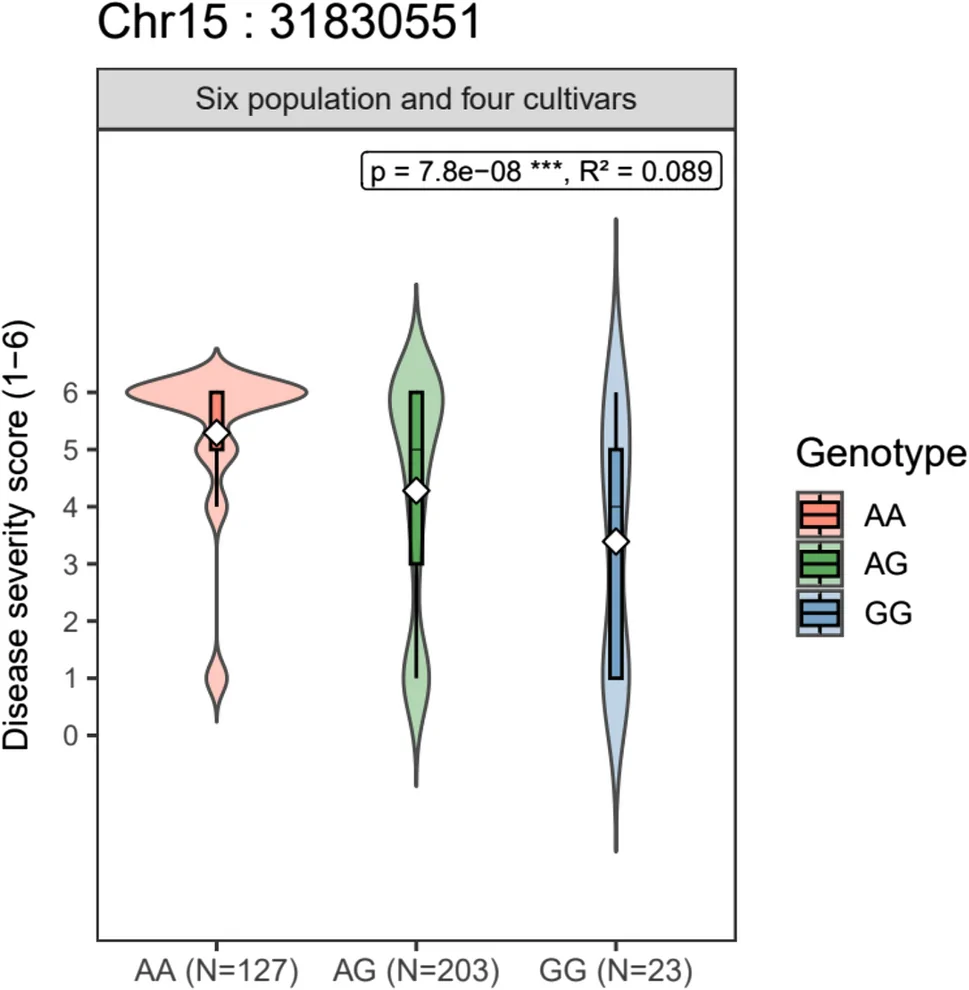
Violin plot showing the distribution of field-derived disease severity scores according to genotype at SNP Chr15:31830551 across six apple F₁ populations and four parental cultivars evaluated in 2019. R² indicates the proportion of variation in field degree-of-infection score explained by the SNP genotype

Analysis of the 2019 field dataset revealed a prominent association peak on chromosome 15 at approximately 31.8 Mb (Fig. 3). The lead SNP at this locus (Chr15:31830551) showed the strongest statistical support, with –log₁₀p values of 11.51 in BLINK and 8.42 in FarmCPU. This SNP is located in an intergenic region proximal to DVH24_021233, which encodes ATP-dependent 6-phosphofructokinase 5 (Table 8). Additional significant associations were detected on chromosome 14 at ~ 28.9 Mb near DVH24_023685, annotated as a probable galacturonosyltransferase 11, and on chromosome 16 at ~ 6.1 Mb within the exon of DVH24_007057, an uncharacterized gene. Both loci were identified using the FarmCPU model. Although the SUPER model identified multiple SNPs exceeding the significance threshold on chromosome 15, only associations supported by BLINK and/or FarmCPU were retained in the final summary, as these models showed more consistent statistical behavior for this dataset. Based on these criteria, Chr15:31830551 represented the most robust association detected in the 2019 field evaluation (Figs. 3 and 4; Table 8).

### GWAS results from the 2020 field dataset

GWAS using the 2020 field degree-of-infection scores identified a distinct set of significant associations relative to 2019. Five SNPs forming a cluster on chromosome 13 between approximately 1.5 and 2.6 Mb exceeded the genome-wide significance threshold (Table 8). These SNPs were detected exclusively by the SUPER model and exhibited –log₁₀p values ranging from approximately 6.3 to 8.1. No corresponding significant associations on chromosome 13 were identified by the other GWAS models applied, including FarmCPU, BLINK, MLM, and MLMM.

### GWAS results in the ‘Gala’ × ‘Jonathan’ population

**Fig. 5:**
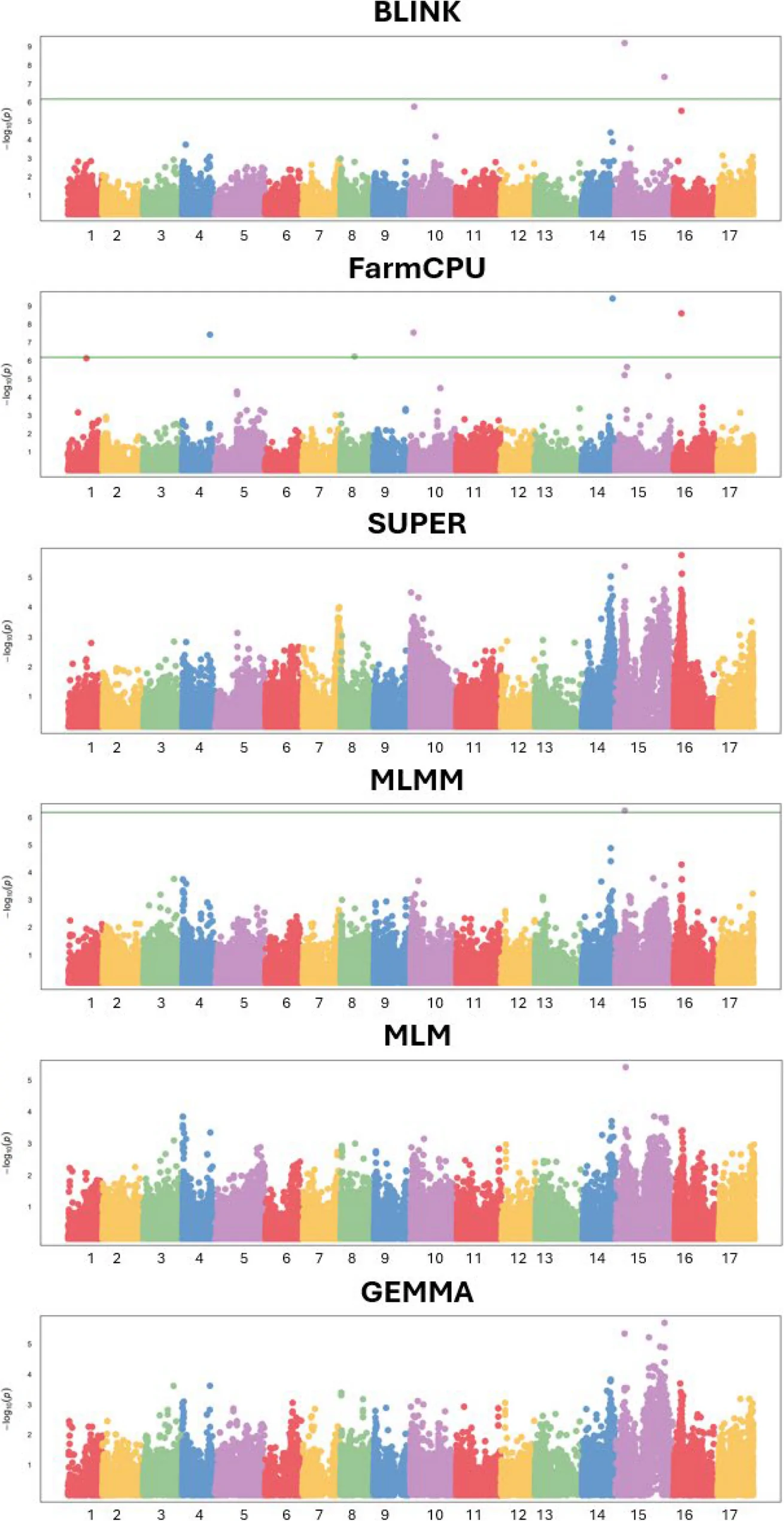
Genome-wide association study (GWAS) results for field-derived anthracnose disease severity scores in the ‘Gala’ × ‘Jonathan’ population in 2020, expressed as degree-of-infection scores on a 1–6 scale, analyzed using five GAPIT models (BLINK, FarmCPU, SUPER, MLMM, and MLM) and one GEMMA model (LMM). The solid green line indicates the Bonferroni-corrected genome-wide significance threshold

Analysis of the ‘Gala’ × ‘Jonathan’ population evaluated in 2020 revealed several genome-wide significant associations (Fig. 5; Table 8). Significant SNPs were identified on chromosomes 10, 14, 15, and 16. The strongest association was detected on chromosome 15 at approximately 9.2 Mb, exceeding the Bonferroni threshold in both the BLINK and MLMM models. Additional associations were observed on chromosome 16 at ~ 7.1 Mb and on chromosome 10 at ~ 3.2 Mb.

### Comparison across years and populations

Comparison of GWAS results across years showed non-overlapping sets of significant loci between the 2019 and 2020 field datasets (Fig. 3; Table 8). The chromosome 15 locus detected in 2019 was not identified in the 2020 analysis, whereas chromosome 13 associations detected in 2020 were absent in the 2019 dataset. Similarly, population-specific analyses revealed additional loci not observed in the combined-population GWAS.

## Discussion

### Field disease severity patterns and cultivar-specific responses

Multi-year field evaluations revealed clear cultivar-level differences in anthracnose disease incidence. ‘Fuji’, ‘Aikanokaori’, ‘Tsugaru’, and ‘Gala’ showed consistently low fruit infection from 2009 to 2011, whereas ‘Jonathan’, ‘Hongro’, and ‘Cripps Pink’ showed higher disease incidence under the same orchard conditions. These patterns were broadly consistent with previous field-based studies identifying ‘Fuji’ as a cultivar with low anthracnose incidence [34]. However, some discrepancies were observed when our standardized field assessments were compared with grower-reported evaluations from the Mid-Atlantic region [35]. Such differences likely reflect regional variation in pathogen populations, climate, disease pressure, and evaluation methods. These observations highlight the importance of standardized, multi-year field evaluations for characterizing anthracnose disease response in apple.

### Environmental modulation of field disease severity

Field observations and previous studies indicate that anthracnose disease severity is strongly influenced by environmental conditions. Under prolonged warm and humid conditions favorable for pathogen infection, genotypes with relatively low disease scores under moderate disease pressure may show increased symptom severity. In the present study, this pattern was evident in 2020, when prolonged rainfall likely increased *Colletotrichum* infection pressure [36, 37]. The resulting shift toward higher disease severity scores underscores the strong influence of genotype-by-environment interactions and supports the need for multi-season field evaluations [38, 39].

### Limitations of controlled inoculation assays for predicting field disease severity

In contrast to orchard performance, controlled inoculation assays did not reliably predict field disease severity. Non-wound fruit inoculation produced minimal lesions in many genotypes that developed clear symptoms under orchard conditions, indicating a disconnect between laboratory and field responses [40]. This discrepancy likely reflects differences in infection routes and environmental stress factors, including micro-injuries, fluctuating humidity, and prolonged surface wetness. While controlled inoculation assays remain useful for preliminary screening, they cannot substitute for field validation under natural infection pressure [39, 41, 42]. In this study, this limitation was also evident at the genetic level: genome-wide significant associations were detected only from field-derived disease severity scores, not from artificial inoculation traits.

### Field-based GWAS identifies a chromosome 15 locus associated with reduced disease severity

In the multi-family GWAS, genome-wide significant SNPs were detected only from field-derived disease severity scores, with the strongest signal observed in the 2019 dataset. The most robust association was located on chromosome 15 at approximately 31.8 Mb. This region is physically distinct from the previously characterized *Rgls/MdTNL1* region on chromosome 15, which is located at approximately 2–5 Mb and has been associated with GLS response [10–12]. Therefore, the Chr15:31.8 Mb locus likely represents an independent disease severity-associated QTL rather than the previously reported *Rgls* locus. The lead SNP was located near DVH24_021233, which encodes an ATP-dependent 6-phosphofructokinase. Because the functional role of this gene in anthracnose disease response remains unclear, it should be considered a preliminary candidate requiring further validation.

### Interpretation limits and the need for further validation

Despite the strong statistical support for the Chr15:31.8 Mb locus in the 2019 dataset, caution is warranted when interpreting causality at the gene level. As is common in GWAS, the significant SNPs identified are likely to be in linkage disequilibrium with the true functional variants rather than being causal themselves. In addition, the relatively sparse marker density inherent to GBS-based genotyping limits the resolution of association peaks, making it difficult to narrow associations to individual genes [43, 44]. Several significant associations identified in this study were also model-specific. In particular, the chromosome 13 signal detected in the 2020 dataset was identified only by the SUPER model and was not consistently supported by the other GWAS models. Therefore, greater emphasis was placed on loci supported by multiple models, while single-model associations were interpreted more cautiously. Moreover, the reduced detectability of this locus under the high disease pressure observed in 2020 suggests that its phenotypic effect may be conditional rather than universally expressed across environments. For these reasons, we interpret the Chr15:31.8 Mb region as a promising disease severity-associated QTL that merits further investigation, rather than as a fully validated causal locus. Additional multi-year validation, higher-density genotyping, and fine-mapping approaches will be required to confirm the stability and functional basis of this association.

## Conclusion

This study identified a chromosome 15 locus associated with reduced field-derived anthracnose disease severity using multi-year phenotyping and GWAS. Disease severity was strongly influenced by environmental conditions, and field-derived phenotypes produced more robust association signals than controlled inoculation assays. These findings highlight the importance of field-based multi-environment evaluations for identifying disease-response loci relevant to apple breeding.

## Supplementary Information

Supplementary Material 1.

Supplementary Material 2.

## Data Availability

The sequencing data generated in this study have been deposited in the NCBI Sequence Read Archive (SRA) under BioProject accession number PRJNA1450802. Phenotypic datasets used for GWAS, including field degree-of-infection scores and artificial inoculation disease severity scores, are provided in Supplementary Table S1.

## Abbreviations

GWAS: Genome-wide association study
GLS: Glomerella leaf spot
SNP: Single nucleotide polymorphism
GBS: Genotyping-by-sequencing
QTL: Quantitative trait locus
G × E: Genotype-by-environment interaction
MLM: Mixed linear model
MLMM: Multi-locus mixed model
LMM: Linear mixed model
PCA: Principal Component Analysis
PDA: Potato dextrose agar
RDA: Rural Development Administration
NIHHS: National Institute of Horticultural & Herbal Science
