# Supplementary material for: Genome-wide association study combined with multi-assay phenotyping identifies a novel anthracnose resistance locus in apple
Source: BMC Plant Biol. 2026 Jun 24;26:1533. doi: 10.1186/s12870-026-09198-y (PMC13548689; doi:10.1186/s12870-026-09198-y)
Supplement: Supplementary file 1 [file 12870_2026_9198_MOESM1_ESM.pdf]

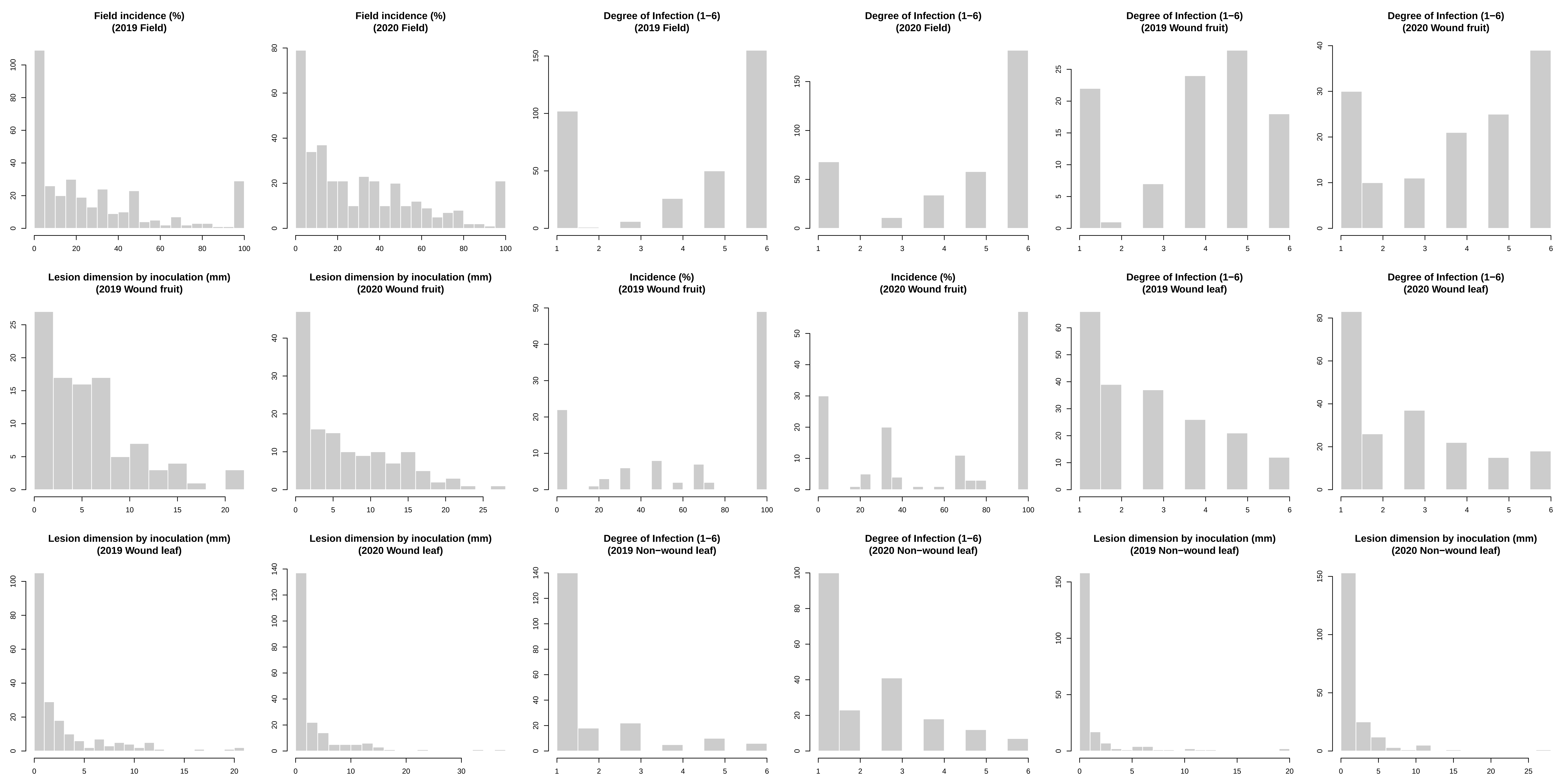
Field incidence (%)
(2019 Field)
Field incidence (%)
(2020 Field)
Degree of Infection (1−6)
(2019 Field)
Degree of Infection (1−6)
(2020 Field)
Degree of Infection (1−6)
(2019 Wound fruit)
Degree of Infection (1−6)
(2020 Wound fruit)
Lesion dimension by inoculation (mm)
(2019 Wound fruit)
Lesion dimension by inoculation (mm)
(2020 Wound fruit)
Incidence (%)
(2019 Wound fruit)
Incidence (%)
(2020 Wound fruit)
Degree of Infection (1−6)
(2019 Wound leaf)
Degree of Infection (1−6)
(2020 Wound leaf)
Lesion dimension by inoculation (mm)
(2019 Wound leaf)
Lesion dimension by inoculation (mm)
(2020 Wound leaf)
Degree of Infection (1−6)
(2019 Non−wound leaf)
Degree of Infection (1−6)
(2020 Non−wound leaf)
Lesion dimension by inoculation (mm)
(2019 Non−wound leaf)
Lesion dimension by inoculation (mm)
(2020 Non−wound leaf)
